# Temporal order of clinical, imaging, and biomarker changes in frontotemporal lobar degeneration–associated syndromes

**DOI:** 10.1002/alz.71722

**Published:** 2026-08-05

**Authors:** Alberto Benussi, Valeria Bracca, Enrico Premi, Valentina Cantoni, Federica Palacino, Aurora Saccavini, Maria Sofia Cotelli, Giuliano Binetti, Rosa Manenti, Antonella Alberici, Roberto Gasparotti, Nicholas J Ashton, Henrik Zetterberg, Kaj Blennow, Roberta Ghidoni, Barbara Borroni

**Affiliations:** ^1^ Neurology Unit Department of Medical, Surgical and Health Sciences University of Trieste Trieste Italy; ^2^ Neurology Unit Hospital Care Department of Medicine Azienda Sanitaria Universitaria Giuliano Isontina Trieste Italy; ^3^ Department of Clinical and Experimental Sciences University of Brescia Brescia Italy; ^4^ Department of Molecular and Translational Medicine University of Brescia Brescia Italy; ^5^ Stroke Unit Azienda Socio Sanitaria Territoriale (ASST) Spedali Civili Brescia Italy; ^6^ Department of Continuity of Care and Frailty ASST Spedali Civili of Brescia Brescia Italy; ^7^ Molecular Markers Laboratory IRCCS Istituto Centro San Giovanni di Dio Fatebenefratelli Brescia Italy; ^8^ Neuropsychology Unit IRCCS Istituto Centro San Giovanni di Dio Fatebenefratelli Brescia Italy; ^9^ Neuroradiology Unit University of Brescia Brescia Italy; ^10^ Department of Psychiatry and Neurochemistry Institute of Neuroscience and Physiology the Sahlgrenska Academy at the University of Gothenburg Mölndal Sweden; ^11^ King's College London London UK; ^12^ NIHR Maudsley Biomedical Research Centre London UK; ^13^ Banner Sun Health Research Institute Sun City Arizona USA; ^14^ Clinical Neurochemistry Laboratory Sahlgrenska University Hospital Mölndal Sweden; ^15^ Department of Pathology and Laboratory Medicine University of Wisconsin School of Medicine and Public Health Madison Wisconsin USA; ^16^ Wisconsin Alzheimer's Disease Research Center University of Wisconsin School of Medicine and Public Health Madison Wisconsin USA; ^17^ Department of Neurodegenerative Disease UCL Institute of Neurology London UK; ^18^ UK Dementia Research Institute at UCL London UK; ^19^ Centre for Brain Research Indian Institute of Science Bangalore India; ^20^ Pitié‐Salpêtrière Hospital Sorbonne University Paris France; ^21^ University of Science and Technology of China and First Affiliated Hospital of USTC Hefei Anhui China

**Keywords:** biomarker cascade, brain atrophy, clinical trial design, disease progression, frontotemporal dementia, frontotemporal lobar degeneration, neurofilament light chain, neuropsychological assessment, plasma GFAP, white matter hyperintensities

## Abstract

**BACKGROUND:**

The temporal sequence of clinical, imaging, and biological changes in sporadic frontotemporal lobar degeneration (FTLD)–associated syndromes remains poorly characterized, and a comprehensive biomarker cascade model is lacking.

**METHODS:**

We developed a data‐driven biomarker cascade model in 489 patients across the FTLD spectrum (211 behaviorial variant frontotemporal dementia [bvFTD], 129 primary progressive aphasia [PPA], 71 corticobasal syndrome [CBS], 66 progressive supranuclear palsy [PSP], and 12 FTD associated with amyotrophic lateral sclerosis [FTD‐ALS]; 1904 patient‐visit observations). Plasma, magnetic resonance imaging (MRI), and clinical biomarkers were modeled using sigmoid trajectories fitted to covariate‐adjusted longitudinal data.

**RESULTS:**

Plasma glial fibrillary acidic protein departed from normality earliest, followed by Trail Making Test Part B (TMT‐B), white matter lesion volume, and neurofilament light chain. Insular atrophy showed the steepest transition among MRI measures; clinical dementia rating dementia staging instrument plus National Alzheimer's Coordinating Center behavior and language domains sum of boxes declined most steeply overall. TMT‐B inflected earliest in bvFTD, whereas insula atrophy dominated in PPA.

**CONCLUSIONS:**

This first data‐driven temporal cascade of multimodal biomarkers in sporadic FTLD‐associated syndromes offers a framework for disease staging and stage‐specific clinical trial design.

## Highlights

- Data‐driven biomarker cascade model for sporadic frontotemporal lobar degeneration (FTLD)
- Plasma glial fibrillary acidic protein (GFAP) shows the earliest model‐estimated departure from normality
- Executive dysfunction (Trail Making Test Part B [TMT‐B]) shows the earliest model‐estimated inflection point
- Insula atrophy exhibits the steepest transition among magnetic resonance imaging (MRI) measures
- Cascade ordering is preserved across cortical and motor syndromes

## 1 BACKGROUND

Frontotemporal lobar degeneration (FTLD)–associated syndromes are progressive neurodegenerative disorders characterized by marked pathological and clinical heterogeneity. Diagnostic criteria are based primarily on presenting clinical features, including the behavioral variant of frontotemporal dementia (bvFTD),^1^ which is associated with early behavioral and executive dysfunction; the primary progressive aphasias (PPAs), characterized by progressive language impairment;^2^ and motor presentations, such as progressive supranuclear palsy (PSP), corticobasal syndrome (CBS), or FTD associated with amyotrophic lateral sclerosis (FTD‐ALS).^3^, ^4^, ^5^

FTLD‐related syndromes are associated with atrophy of the frontal and temporal lobes, with the insular cortex proposed as a key epicenter or driving the spread of pathology.^6^, ^7^, ^8^ Concomitantly, white matter hyperintensities (WMHs) have been variably described in both genetic and sporadic forms of the disease and have been associated with cognitive performance.^9^, ^10^, ^11^, ^12^ FTLD‐related syndromes lack well‐established disease‐specific biological markers. Nevertheless, markers of neurodegeneration, such as plasma neurofilament light chain (NfL), and markers of astrogliosis, including plasma glial fibrillary acidic protein (GFAP), have been consistently shown to be increased.^13^, ^14^, ^15^ Clinical, imaging, and biological alterations are readily detectable during symptomatic stages of FTLD‐related syndromes; however, abnormalities associated with prodromal disease phases, referred to as mild cognitive, behavioral, and/or motor impairment (MCBMI),^16^, ^17^, ^18^ remain incompletely characterized.

**RESEARCH IN CONTEXT**

1. **Systematic review**: We searched PubMed using the terms “frontotemporal dementia”, “frontotemporal lobar degeneration”, “biomarker”, “progression”, and “temporal ordering” from database inception to December 31, 2025, with no language restrictions. Existing biomarker cascade models in neurodegeneration have been developed primarily for Alzheimer's disease, providing a hypothetical temporal sequence of amyloid, tau, neurodegeneration, and cognitive markers. For frontotemporal dementia, recent studies using event‐based and data‐driven modeling have characterized biomarker ordering in genetic forms (*progranulin* ‐ *GRN*, *microtubule associated protein tau* ‐ *MAPT*, *chromosome 9 open reading frame 72* ‐ *C9orf72*), but no comprehensive cascade model exists for sporadic frontotemporal lobar degeneration [FTLD]–associated syndromes integrating clinical, imaging, and plasma biomarkers.
2. **Interpretation**: This study provides the first data‐driven biomarker cascade model for sporadic FTLD‐associated syndromes, integrating 10 biomarkers across three domains (plasma, magnetic resonance imaging [MRI], neuropsychological) in 489 patients with up to five longitudinal assessments. We demonstrate that plasma glial fibrillary acidic protein (GFAP) and executive dysfunction (Trail Making Test Part B [TMT‐B]) are the earliest detectable changes, followed by white matter alterations, regional cortical atrophy centered on the insular cortex, and global clinical decline. Subgroup analyses reveal phenotype‐specific cascade differences between behavioral variant frontotemporal dementia (bvFTD) and primary progressive aphasia (PPA).
3. **Future directions**: These findings provide a temporal framework for sporadic FTLD that parallels established Alzheimer's disease models, supporting the development of stage‐specific diagnostic criteria and clinical trial endpoints. The early departure of GFAP and TMT‐B suggests that these biomarkers may serve as targets for early detection and intervention studies in prodromal FTLD.

Whereas Alzheimer's disease (AD) progression models propose a temporally ordered sequence of biomarker abnormalities,^19^, ^20^ the marked heterogeneity of FTLD has consistently hindered a straightforward staging of the disease from MCBMI to overt symptomatic phases, and little is known about the ontogeny of biomarkers and clinical changes. To date, a disease progression model for FTLD‐related syndromes has not been established. Such a model could, in turn, advance our understanding of disease mechanisms, support the development of diagnostic and prognostic prediction tools, characterize early FTLD‐related changes, and inform the design of clinical trials.^21^

It might be hypothesized that clinical symptoms emerge only after antecedent biological changes with overt structural atrophy representing a later‐stage consequence of this pathological cascade. However, the temporal ordering of these processes remains unresolved. It remains unclear whether astroglia activation precedes or follows neurodegenerative marker alterations, whether atrophy in disease‐relevant regions occurs before or after the emergence of WMHs, and what the temporal relationship is between changes in biological and imaging markers.

These above observations prompted the present study. We leveraged a large cohort of 489 individuals with FTLD‐associated syndromes, comprising more than 1900 observations, and developed a joint model integrating clinical, imaging, and biological data to characterize the temporal ordering of pathological and clinical changes.

## 2 METHODS

***Participants***. This retrospective longitudinal study recruited participants recruited from the University of Brescia and IRCCS Fatebenefratelli, Italy. Patients met the current clinical criteria for an FTLD‐associated syndrome, including the bvFTD,^1^ the agrammatic/nonfluent variant of PPA (nfvPPA) or semantic variant of PPA (svPPA),^2^ PSP,^3^ CBS, ^4^ or FTD‐ALS.^5^

All participants underwent an extensive neuropsychological evaluation, following standard procedures, as reported previously.^22^, ^23^ Disease severity was assessed with the clinical dementia rating (CDR) dementia staging instrument plus National Alzheimer's Coordinating Center (NACC) behavior and language domains (CDR plus NACC FTLD) sum of boxes (CDR‐SoB).^24^ Disease duration was estimated from symptom onset, determined according to patient and/or caregiver report upon discussion with a specialized neurologist, and date of assessment.

Brain magnetic resonance imaging (MRI) scans were performed on all participants. For a subgroup of participants, the diagnosis was confirmed with amyloid markers, including cerebrospinal fluid (CSF) total tau (t‐tau), phosphorylated tau 181 (p‐tau181), and amyloid beta (Aβ)1–42 determinations, or amyloid positron emission tomography (PET) imaging with [^18^F]‐florbetapir or [^18^F]‐flutemetamol, ruling out an AD diagnosis, as reported previously.^25^ When diagnostic confidence in selected cases was not satisfactory, additional procedures, including brain fluorodeoxyglucose PET, were used. Furthermore, in familial cases (defined by the presence of at least one dementia case among the first‐degree relatives) and early onset sporadic cases, genetic screening for monogenic forms of FTLD‐associated syndromes was performed.^26^ Genetic testing identified pathogenic variants in 104 of the 489 patients (*progranulin* ‐ *GRN*, *n* = 61; *chromosome 9 open reading frame 72* ‐ *C9orf72*, *n* = 29; *TAR DNA‐binding protein 43* ‐ *TARDBP*, *n* = 8; *microtubule associated protein tau* ‐ *MAPT*, *n* = 4; *sequestosome 1* ‐ *SQSTM1*, *n* = 1; *fused in sarcoma* ‐ *FUS*, *n* = 1) (see Table S1); analyses were performed on both the full cohort and in non‐carriers.

Full written informed consent was obtained from all subjects according to the Declaration of Helsinki. The study protocol was approved by the Brescia Ethics Committee.

***Cognitive and behavioral assessment***. At baseline and at each cognitive and behavioural assessment, patients underwent a standardized neuropsychological battery. For the purposes of the present study, we considered the Trail Making Test Part A (TMT‐A) tapping processing speed and sustained attention, TMT Part B (TMT‐B)^27^, ^28^ tapping high order executive functions, and the Frontal Behavioural Inventory (FBI)^28^ to assess behavioral disturbances.

***MRI acquisition and analyses***. Three‐dimensional T1‐weighted magnetization‐prepared rapid acquisition with gradient echo (MPRAGE) MRI scans were acquired using either a 1.5 Tesla (Siemens Avanto and Siemens Symphony, Erlangen, Germany) or a 3 Tesla scanner (Siemens Skyra, Erlangen, Germany). All T1‐weighted and fluid‐attenuated inversion recovery (FLAIR) images were visually inspected and excluded from further analysis in case of excessive motion blurring or artifacts.

T1‐weighted images were processed using the voxel‐based morphometry (VBM) pipeline implemented in Computational Anatomy Toolbox (CAT12 v.1742) for Statistical Parametric Mapping 12 (SPM12), including tissue segmentation, normalization to standard Montreal Neurological Institute (MNI) space, modulation, and smoothing, to obtain estimates of regional gray matter (GM) volume.^29^, ^30^ Normalized and modulated images were smoothed with an 8 × 8 × 8 mm full width at half maximum (FWHM) Gaussian kernel. CAT12 was also used to extract GM measures. Frontal and insula region of interest (ROI) masks were defined using the Automated Anatomical Atlas (AAL) in Wake Forest University PickAtlas,^31^ and resliced to match the VBM resolution using nearest‐neighbor interpolation. Frontal and insula GM volumes within the ROIs were then extracted using the REX toolbox for SPM12, implemented within the CONN toolbox (https://web.conn‐toolbox.org). The VBM images were used as the source images, whereas the ROI masks as reference ROIs. The number of voxels for every region was extracted with an ROI‐level analysis without conjunction masking or global scaling. For each participant, ROI volume was then calculated with the number of voxels in the mask multiplied by the single‐voxel volume. Mean values were extracted separately for left and right frontal and insular regions and were subsequently averaged to obtain a single regional measure. GM, frontal, and insular volumes were used as proxies for atrophy in the subsequent statistical analyses.

FLAIR scans were processed with the lesion prediction algorithm,^32^, ^33^ implemented in the Lesion Segmentation Toolbox (LST, v.3.0.0) (www.statistical‐modelling.de/lst.html) for SPM12. We considered the total WMH volume (expressed in milliliters [mL]) as a measure of WM burden.

***Plasma biomarkers***. Plasma samples were collected according to standard procedures and stored at −80°C until use. Biomarker measurements were carried out at the University of Gothenburg, Sweden. Plasma NfL and GFAP concentrations were measured using a commercial Single molecule array (Simoa, Quanterix, Billerica, MA) according to the manufacturer's instructions, as published previously.^34^, ^35^ Samples were run as singlicates on a Quanterix Simoa‐HD‐X platform in one round of experiments using one batch of reagents. Intra‐ and inter‐assay coefficients of variation (<15%) were monitored using internal quality control samples with clinically relevant high and low NfL and GFAP concentrations.

***Statistical analysis***. The temporal ordering of multimodal biomarkers across the FTLD continuum was estimated in a pooled spectrum cohort of 489 patients with up to five longitudinal assessments (1904 patient‐visit observations). The primary estimand was the biomarker‐specific inflection time θ_k_, defined as the disease duration at which the modeled trajectory reached 50% of its estimated transition on a bounded abnormality scale, with smaller θ_k_ indicating earlier transition. Three normalization frameworks were prespecified. The primary framework standardized all biomarkers against an internal reference defined by the first quartile of baseline CDR‐SoB, representing the mildest symptomatic patients rather than healthy controls, using regression‐based demographic correction for neuropsychological measures and mean–standard deviation (SD) normalization for the remainder. Calibrated secondary frameworks incorporated healthy‐control–based normalization for plasma biomarkers (NfL, GFAP) and externally derived age‐, sex‐, and education‐adjusted normative z‐scores for TMT‐A and TMT‐B. Covariate‐adjusted longitudinal trajectories were estimated with mixed‐effects models including restricted cubic splines for disease duration, age, sex, phenotype, and a phenotype‐by‐time interaction term. Marginal trajectories were rescaled to [0, 1] and summarized by biomarker‐specific logistic sigmoids. Monotonicity of the marginal trajectories was checked over the analysis window: 8 of the 10 biomarkers were monotonic, whereas GFAP and NfL were flagged as non‐monotonic (a minor deviation, with a maximum decrease <0.001 in normalized units per time step), so their sigmoid parameters are interpreted as descriptive summaries rather than evidence of a strictly sigmoidal transition. Cascade ordering was determined by ranked θ̂_k_ values. Uncertainty was quantified using a patient‐level cluster bootstrap (1000 iterations) repeating the full pipeline, with pairwise precedence probabilities computed across all biomarker pairs. Prespecified sensitivity analyses assessed robustness to symptom‐onset perturbation, normalization framework, spline flexibility, covariate parameterization, random effects structure, sigmoid regularization, and percentile anchors. Full methodological details are provided in the **Supplementary Methods**. Analyses were conducted in Python 3.10 using NumPy, pandas, statsmodels for mixed effects and regression models, SciPy for bounded optimization, and matplotlib for visualization.

## 3 RESULTS

A total of 489 patients with a clinical diagnosis within the FTLD spectrum were included, comprising 211 bvFTD (43.1%), 92 non‐fluent variant (nfvPPA, 18.8%), 37 semantic variant (svPPA, 7.6%), 71 CBS (14.5%), 66 PSP (13.5%), and 12 FTD‐ALS (2.5%). The cohort included 223 women (45.6%) and 263 men (53.8%), with a mean age at symptom onset of 63.1 ± 8.2 years and a mean education of 9.1 ± 4.3 years. Mean disease duration at baseline was 2.9 ± 2.0 years. Of the 489 patients, 133 (27.2%) contributed one visit, 126 (25.8%) contributed two visits, 96 (19.6%) contributed three visits, 110 (22.5%) contributed four visits, and 24 (4.9%) contributed five visits, for a total of 1904 patient‐visit observations (the numbers of available observations for each biomarker, overall and by epoch, are reported in see Table S2). Mean follow‐up duration among patients with more than one visit (*n* = 451) was 2.0 ± 2.0 years. Demographic and clinical characteristics of the study sample are summarized in Table 1.

**TABLE 1:** Demographic and clinical characteristics of the study sample.

Characteristic	All FTLD (*n * = 489)	bvFTD (*n * = 211)	PPA (*n * = 129)
Sex, female, *n* (%)	223 (45.6)	78 (37.0)	81 (62.8)
Age at symptom onset, years	63.1 ± 8.2	62.1 ± 7.9	62.4 ± 8.6
Education, years	9.1 ± 4.3	9.1 ± 4.2	10.4 ± 4.3
Disease duration at baseline, years	2.9 ± 2.0	2.9 ± 2.0	2.8 ± 2.0
MMSE at baseline	21.7 ± 6.4	21.9 ± 5.4	18.4 ± 8.1
CDR‐SoB at baseline, median (IQR)	3.2 (1.5–6.0)	4.0 (1.5–6.0)	4.0 (2.5–6.0)
Number of visits, n (%)			
1	133 (27.2)	65 (30.8)	36 (27.9)
2	126 (25.8)	59 (28.0)	30 (23.3)
3	96 (19.6)	27 (12.8)	34 (26.4)
4	110 (22.5)	45 (21.3)	26 (20.2)
5	24 (4.9)	15 (7.1)	3 (2.3)
Follow‐up duration, years	2.4 ± 2.0	2.5 ± 2.3	2.1 ± 2.0

*Note*: Values are mean ± SD unless otherwise specified. PPA includes non‐fluent variant PPA (*n * =  92) and semantic variant PPA (*n * =  37).

Abbreviations: bvFTD, behavioural variant frontotemporal dementia; FTLD, frontotemporal lobar degeneration associated syndromes; PPA, primary progressive aphasia. N, number; MMSE, Mini‐Mental State Examination, CDR‐SoB, clinical dementia rating (CDR) dementia staging instrument plus National Alzheimer's Coordinating Center (NACC) behavior and language domains (CDR plus NACC FTLD) sum of boxes; IQR, interquartile range.

### 3.1 Biomarker cascade ordering in the pooled FTD cohort

**FIGURE 1:**
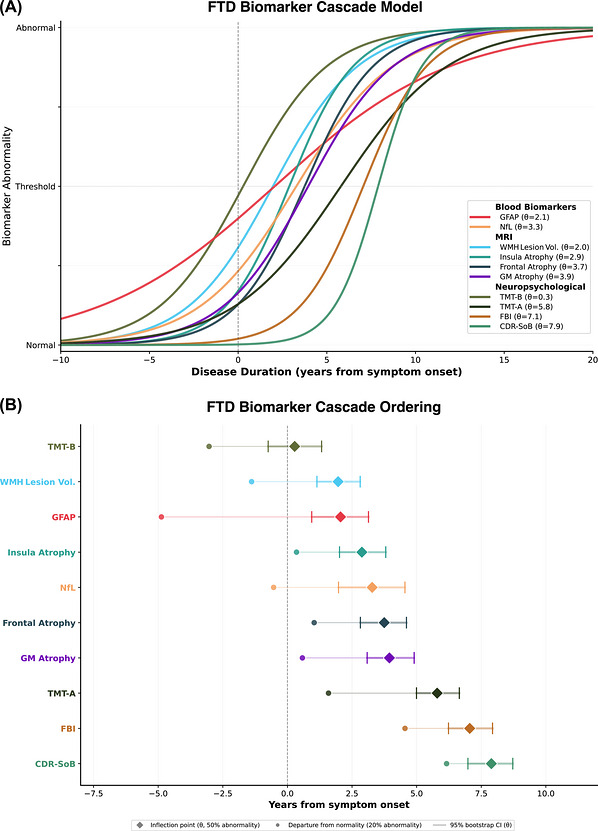
Biomarker cascade model in the pooled FTD cohort. (A) Estimated sigmoid trajectories for 10 biomarkers as a function of disease duration (years from symptom onset). Each curve represents the modeled transition from normal to fully abnormal, with the inflection point (θ) indicating when each biomarker reaches 50% of its total change. Biomarkers are grouped by domain: blood biomarkers (GFAP, NfL), MRI measures (WMH volume, insula atrophy, frontal atrophy, GM atrophy), and neuropsychological/clinical measures (TMT‐B, TMT‐A, FBI, CDR‐SoB). (B) Forest plot showing the estimated inflection points (θ, diamonds) with 95% bootstrap CIs for each biomarker, ordered by cascade position. Circles indicate the estimated time of departure from normality, defined as the time at which each biomarker reaches 20% of its total change. The dashed vertical line indicates symptom onset (*t* = 0).CI,  confidence interval; CDR‐SoB, clinical dementia rating plus National Alzheimer's Coordinating Center (NACC) behavior and language domains (CDR plus NACC FTLD) sum of boxes; FBI, Frontal Behavioral Inventory; GFAP, plasma glial fibrillary acidic protein; GM, gray matter; MRI, magnetic resonance imaging; NfL, plasma neurofilament light chain; TMT, Trail Making Test; WMH, white matter hyperintensity.

**FIGURE 2:**
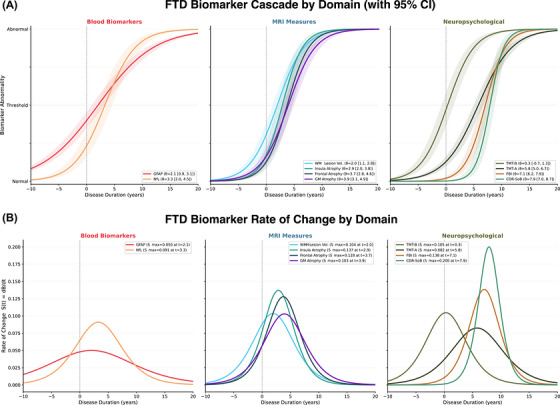
Domain‐specific biomarker trajectories and rates of change in the pooled FTD cohort. (A) Sigmoid trajectories grouped by biomarker domain (blood biomarkers, magnetic resonance imaging measures, neuropsychological/clinical), with 95% bootstrap confidence intervals (shaded areas). Within each domain, biomarkers are ordered by their inflection point (θ), with values and 95% CIs shown in the legend. (B) Instantaneous rate of change (S(t)  =  dB/dt) for each biomarker, representing the first derivative of the sigmoid curves. Peak rate of change (S_max_) and its timing are indicated in the legend. Higher peaks indicate steeper transitions; earlier peaks indicate more rapid early deterioration. CDR‐SoB, clinical dementia rating (CDR) dementia staging instrument plus National Alzheimer's Coordinating Center (NACC) behavior and language domains (CDR plus NACC FTLD) sum of boxes; CI, confidence interval; FBI, Frontal Behavioral Inventory; GFAP, plasma glial fibrillary acidic protein; GM, gray matter; NfL, plasma neurofilament light chain; TMT, Trail Making Test; WMH, white matter hyperintensity.

The estimated biomarker cascade ordering in the pooled FTLD‐associated syndromes cohort is shown in Figure 1A and Figure 2A. Plasma GFAP was the earliest biomarker to depart from normality, owing to its gradual, model‐estimated transition extending into the pre‐symptomatic window. This was followed by TMT‐B, WMH volume, and plasma NfL. Insula atrophy, frontal atrophy, and GM atrophy transitioned subsequently, followed by TMT‐A. FBI and CDR‐SoB were the last biomarkers to depart from normality.

The robustness of this ordering was supported by the pairwise precedence probability matrix, in which 41 of the 45 biomarker pairs (91%) showed a precedence probability ≥0.80 (see Figure S1). The ordering was also stable across all prespecified sensitivity analyses, including symptom‐onset perturbation, normalization framework, spline flexibility, and sigmoid regularization (see Figure S2). Adjusting the MRI biomarkers for scanner field strength likewise left the ordering unchanged (Spearman ρ = 0.98), with differences confined to small shifts (<0.7 years) among the closely spaced cortical atrophy measures.

The inflection points (θ), representing the time at which each biomarker reaches 50% of its total change, showed a partially different ordering owing to differences in transition steepness: TMT‐B had the earliest inflection point, followed by WMH volume, GFAP, insula atrophy, NfL, frontal atrophy, GM atrophy, TMT‐A, FBI, and CDR‐SoB (Figure 1B).

The peak rate of change (S_max_), representing the fastest instantaneous rate of biomarker change, varied substantially across biomarkers (Figure 2B). CDR‐SoB exhibited the steepest transition of any biomarker (S_max _= 0.200), followed by FBI (S_max _= 0.138) and insula atrophy (S_max _= 0.137). Frontal atrophy (S_max _= 0.128), TMT‐B (S_max _= 0.105), WMH volume (S_max _= 0.104), and GM atrophy (S_max _= 0.103) showed intermediate rates of change. NfL (S_max _= 0.091), TMT‐A (S_max _= 0.082), and GFAP (S_max _= 0.050) exhibited the most gradual transitions.

In a sensitivity analysis, excluding the 104 pathogenic variant carriers did not materially change the cascade ordering (Spearman ρ = 0.93; see Table S3 and Figure S3).

### 3.2 Temporal epochs of biomarker abnormality

**FIGURE 3:**
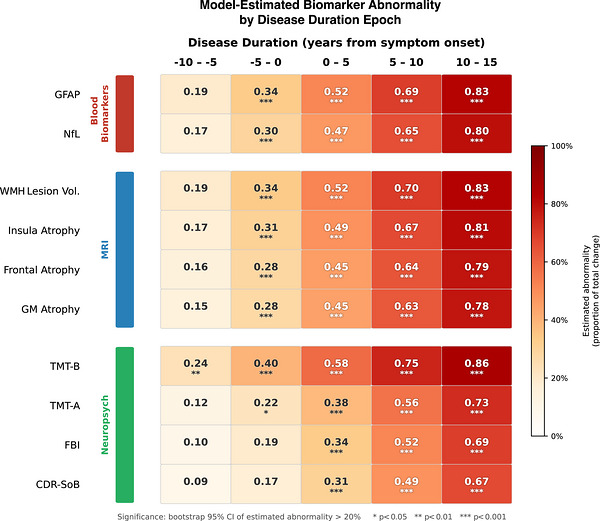
Model‐estimated biomarker abnormality by disease duration epoch. Heatmap showing the estimated proportion of total change (sigmoid value) for each biomarker across 5‐year epochs from –10 to +15 years relative to symptom onset. Color intensity reflects the degree of estimated abnormality (0%  =  normal, 100%  =  fully abnormal). Significance levels are derived from bootstrap 95% CIs of estimated abnormality exceeding 20%: * *p*  <  0.05, ** *p * <  0.01, *** *p * <  0.001. Biomarkers are grouped by domain: blood biomarkers, MRI measures, and neuropsychological/clinical measures. CDR‐SoB, clinical dementia rating (CDR) dementia staging instrument plus National Alzheimer's Coordinating Center (NACC) behavior and language domains (CDR plus NACC FTLD) sum of boxes; CI, confidence interval; FBI, Frontal Behavioral Inventory; GFAP = plasma glial fibrillary acidic protein; GM, gray matter; MRI, magnetic resonance imaging; NfL, plasma neurofilament light chain; TMT, Trail Making Test; WMH, white matter hyperintensity.

To characterize when individual biomarkers became significantly abnormal across the disease course, model‐estimated sigmoid values were evaluated in 5‐year epochs from 10 years before to 15 years after symptom onset (Figure 3). In the earliest epoch (−10 to −5 years), TMT‐B was the only biomarker showing significant abnormality (*p *< 0.01). Between −5 and 0 years, most biomarkers reached significance: all blood biomarkers (GFAP, NfL), all MRI measures (WMH volume, insula atrophy, frontal atrophy, GM atrophy), and both TMT‐B and TMT‐A showed significant abnormality (*p *< 0.05 to *p *< 0.001), whereas FBI and CDR‐SoB had not yet reached the significance threshold. In the 0–5 years epoch following symptom onset, all 10 biomarkers had reached significant abnormality (*p *< 0.001). Because most participants were assessed at or after symptom onset, this ordering reflects the model‐derived temporal position of covariate‐adjusted trajectories; estimates in the pre‐symptomatic window are extrapolations constrained by the parametric (sigmoid) form rather than direct observations.

### 3.3 Subgroup analysis: bvFTD

**FIGURE 4:**
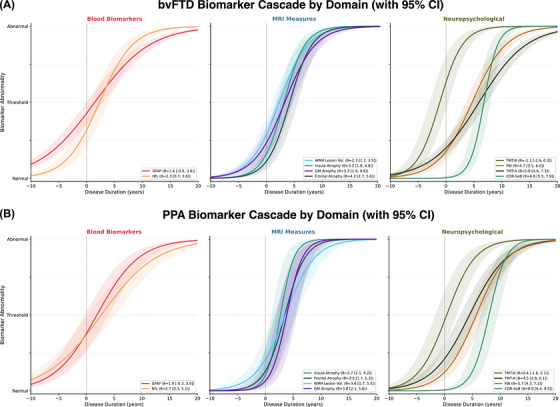
Domain‐specific biomarker cascade trajectories in bvFTD and PPA subgroups. (A) bvFTD subgroup (*n*  =  211): sigmoid trajectories grouped by biomarker domain (blood biomarkers, MRI measures, neuropsychological/clinical), with 95% bootstrap confidence intervals (shaded areas). Inflection points (θ) and 95% CIs are shown in each panel legend. (B) PPA subgroup (*n * =  129, comprising 92 non‐fluent variant PPA and 37 semantic variant PPA): same format as A. bvFTD, behavioral variant frontotemporal dementia; CDR‐SoB, Clinical Dementia Rating (CDR) Dementia Instrument plus National Alzheimer's Coordinating Center (NACC) behavior and language domains (CDR plus NACC FTLD) sum of boxes; CI, confidence interval; FBI, Frontal Behavioral Inventory; GFAP, plasma glial fibrillary acidic protein; GM, gray matter; NfL, plasma neurofilament light chain; PPA, primary progressive aphasia; TMT, Trail Making Test; WMH, white matter hyperintensity.

**FIGURE 5:**
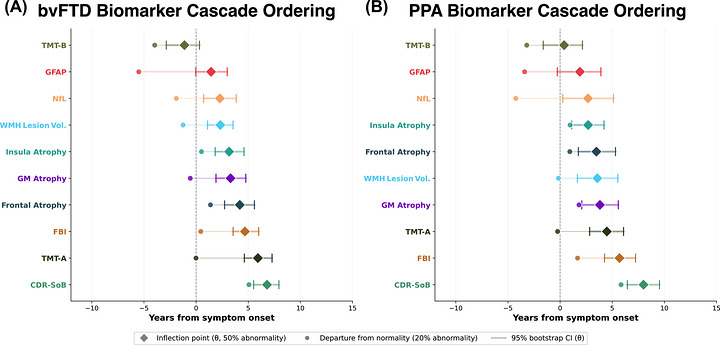
Biomarker cascade ordering in bvFTD and PPA subgroups. **(A)** bvFTD subgroup: forest plot showing estimated inflection points (θ, diamonds) with 95% bootstrap CIs for each biomarker, ordered by cascade position. Circles indicate the estimated time of departure from normality (20% of total change). The dashed vertical line indicates symptom onset (*t* = 0). **(B)** PPA subgroup: same format as A. bvFTD, behavioral variant frontotemporal dementia; bvFTD, behavioral variant frontotemporal dementia; CDR‐SoB, clinical dementia rating (CDR) dementia staging instrument plus National Alzheimer's Coordinating Center (NACC) behavior and language domains (CDR plus NACC FTLD) sum of boxes; CI, confidence interval; FBI ,Frontal Behavioral Inventory; GFAP, plasma glial fibrillary acidic protein; GM, gray matter; NfL, plasma neurofilament light chain; PPA, primary progressive aphasia; TMT, Trail Making Test; WMH, white matter hyperintensity.

**FIGURE 6:**
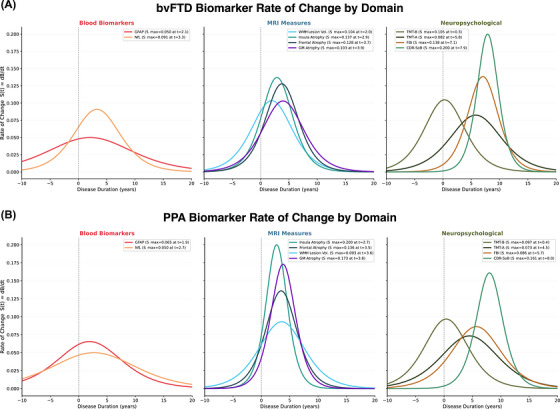
Domain‐specific biomarker rates of change in bvFTD and PPA subgroups. **(A)** bvFTD subgroup: instantaneous rate of change (S(t)  =  dB/dt) for each biomarker grouped by domain. Peak rate of change (S_max_) and its timing are indicated in the legend. **(B)** PPA subgroup: same format as A. Higher peaks indicate steeper transitions. bvFTD, behavioral variant frontotemporal dementia; CDR‐SoB, clinical dementia rating (CDR) plus National Alzheimer's Coordinating Center (NACC) behavior and language domains (CDR plus NACC FTLD) sum of boxes; FBI, Frontal Behavioral Inventory; GFAP, plasma glial fibrillary acidic protein; GM, gray matter; NfL, plasma neurofilament light chain; PPA, primary progressive aphasia; TMT, Trail Making Test; WMH, white matter hyperintensity.

In the bvFTD subgroup, the cascade ordering was broadly consistent with the pooled FTLD analysis, although notable differences emerged (Figure 4A). GFAP was again the earliest biomarker to depart from normality, followed by TMT‐B, NfL, and WMH volume. Among MRI measures, GM volume and insula atrophy preceded frontal atrophy. FBI preceded TMT‐A in departure from normality, and CDR‐SoB was the last biomarker to transition. The inflection points showed a partially different ordering: TMT‐B, GFAP, NfL, WMH volume, insula atrophy, GM atrophy, frontal atrophy, FBI, TMT‐A, and CDR‐SoB (see Figure 5A). The peak rates of change showed that CDR‐SoB had the steepest transition (S_max _= 0.200), followed by insula atrophy (S_max _= 0.131), frontal atrophy (S_max _= 0.123), and TMT‐B (S_max _= 0.122). WMH volume (S_max _= 0.097), GM atrophy (S_max _= 0.090), NfL (S_max _= 0.083), and FBI (S_max _= 0.082) showed intermediate rates, whereas TMT‐A (S_max _= 0.058) and GFAP (S_max _= 0.050) exhibited the most gradual transitions (Figures 6A).

### 3.4 Subgroup analysis: PPA

In the PPA subgroup (nfvPPA and svPPA combined), the cascade showed an overall pattern similar to that of the pooled FTLD analysis, although several phenotype‐specific differences were observed (Figure 4B). Plasma GFAP and NfL were the earliest biomarkers to depart from normality, followed by TMT‐B. Among MRI measures, insula atrophy and frontal atrophy preceded WMH volume and GM atrophy. In contrast to bvFTD, WMH volume transitioned somewhat later in PPA. TMT‐A and FBI preceded CDR‐SoB, which was the last biomarker to depart from normality. The inflection points showed the following ordering: TMT‐B, GFAP, NfL, insula atrophy, frontal atrophy, WMH volume, GM atrophy, TMT‐A, FBI, and CDR‐SoB (see Figure 5B). In contrast to the pooled FTLD and bvFTD analyses, insula atrophy exhibited the steepest transition in PPA (S_max _= 0.200), followed by GM atrophy (S_max _= 0.173) and CDR‐SB (S_max _= 0.161). Frontal atrophy (S_max _= 0.136), TMT‐B (S_max _= 0.097), and WMH volume (S_max _= 0.093) showed intermediate rates. FBI (S_max _= 0.086), TMT‐A (S_max _= 0.073), GFAP (S_max _= 0.065), and NfL (S_max _= 0.050) exhibited the most gradual transitions (Figures 6B).

### 3.5 Subgroup analysis: cognitive versus motor‐predominant syndromes

To examine whether the cascade biomarkers differ between the cognitive/cortical and the motor‐predominant syndromes, we compared the cortical group (bvFTD and PPA; *n* = 340) with the motor‐predominant group (CBS, PSP, and FTD‐ALS; *n* = 149). At comparable disease duration, and after adjustment for age and sex, plasma GFAP and NfL, WM lesion volume, and TMT performance did not differ between the two groups, whereas frontal and insular GM volume, FBI, and CDR‐SB indicated significantly greater abnormality in the cortical syndromes (Figure S4).

In a complementary sensitivity analysis, there was no significant change in the cascade ordering when the model was re‐estimated in the cortical phenotypes or in the motor phenotypes compared with the full model (Spearman ρ = 0.92 and 0.86, respectively).

## 4 DISCUSSION

In this study, we developed a data‐driven biomarker cascade model integrating clinical, neuroimaging, and plasma biomarker data from 489 patients across the FTLD spectrum with up to five longitudinal assessments. To our knowledge, this represents the first characterization of the temporal ordering of multimodal biomarker abnormalities in FTLD‐associated syndromes. The conceptual framework extends the hypothetical model of dynamic biomarkers originally proposed for AD,^19^, ^20^ which postulated a temporally ordered cascade from amyloid to neurodegeneration to clinical symptoms. Our findings suggest that also FTLD‐associated syndromes follow a discernible, model‐derived temporal ordering, in which astrogliosis and executive dysfunction precede structural atrophy, which in turn precedes global clinical decline. Unlike AD, the FTLD cascade is characterized by overlapping transitions across biomarker classes, with no single class plateauing before the next becomes abnormal.

These timing relationships are described by three complementary metrics, and distinguishing them clarifies the cascade. The departure from normality denotes the first detectable deviation from the reference range (operationalized as 20% of the total modeled change) and is therefore most sensitive to gradually rising markers; the inflection time (θ) denotes the midpoint of the transition (50% of the total change) and was the prespecified primary metric for cascade ordering, being the most stable summary of a biomarker's central timing and largely insensitive to the threshold chosen to define abnormality; and the peak rate of change (S_max_) denotes the phase of fastest, most abrupt change. Discrepancies between these metrics are informative rather than contradictory. Accordingly, the cascade is defined primarily by the inflection time, with the departure‐from‐normality and peak‐rate metrics providing complementary information on the earliest detectable signal (relevant to early detection) and the window of most rapid change (relevant to staging and trial monitoring), respectively.

Plasma GFAP, a marker of reactive astrogliosis, was the earliest biomarker to depart from normality, owing to its gradual transition dynamics. Its low sigmoid steepness indicates a protracted, model‐estimated transition extending before symptom onset, consistent with the hypothesis that astrogliosis is a slowly evolving process preceding structural degeneration. We note, however, that the marginal trajectories of GFAP and NfL were flagged as non‐monotonic in the mixed‐effects fit; although this deviation was minor, the early position of GFAP, which derives mainly from its gradual departure from normality rather than from a sharp inflection, should be interpreted with corresponding caution and confirmed in prospective studies. Elevated plasma GFAP has been previously demonstrated in progranulin‐associated FTD, with levels correlating with brain atrophy rates and disease severity,^36^ and similar patterns have been observed in AD, where GFAP rises before NfL, suggesting that astrocytic activation may precede overt neurodegeneration across neurodegenerative diseases.^37^ Plasma NfL, a marker of neuroaxonal damage, followed a similar gradual trajectory, departing from normality after GFAP but before most MRI measures. Previous studies have demonstrated elevated NfL in prodromal genetic and sporadic FTD, where it was the strongest predictor of progression to fully symptomatic disease.^38^, ^39^, ^40^, ^41^, ^42^

Among all biomarkers, TMT‐B had the earliest inflection point (θ = 0.3 years), and was the only biomarker reaching significant abnormality in the −10 to −5 year epoch, indicating that, in the model, executive dysfunction was estimated to become abnormal several years before symptom onset. The dissociation between TMT‐B (early inflection) and TMT‐A (late inflection) suggests that higher‐order executive functions are impaired well before basic processing speed. Previous work in presymptomatic genetic FTD carriers has demonstrated cognitive impairment emerging ≈5 years before expected symptom onset, with TMT‐B abnormalities consistently preceding TMT‐A across disease epochs.^43^, ^44^, ^45^ The early TMT‐B abnormality supports the concept of mild cognitive, behavioral, and motor impairment (or MCBMI) as a recognizable prodromal stage in which subtle executive dysfunction may serve as one of the earliest clinical indicators.^17^, ^18^, ^40^

Among neuroimaging measures, insula atrophy exhibited the steepest transition in both the pooled FTD and PPA analyses, consistent with the proposed role of the insular cortex as a key epicenter of neurodegeneration from which pathology spreads through the brain's connectome.^8^, ^46^, ^47^ Insular GM reduction has been identified as one of the earliest structural changes in presymptomatic genetic FTD carriers.^44^, ^45^, ^48^ WMH volume transitioned before most GM measures, in line with reports of increased WMHs years before symptom onset in genetic FTD.^9^, ^44^, ^49^, ^50^ CDR‐SoB and FBI were the last biomarkers to transition, confirming that clinical deterioration is a late but steep event in the cascade.

Subgroup analyses revealed phenotype‐specific cascade differences. In bvFTD, TMT‐B showed a markedly early inflection point, preceding all other biomarkers, consistent with the early executive dysfunction that defines this phenotype. In PPA, insula atrophy exhibited the steepest transition of any biomarker, and CDR‐SB had a later inflection point than in bvFTD, consistent with the slower functional decline typically observed in language‐predominant variants.

A direct comparison between the cognitive/cortical and motor‐predominant syndromes further clarified which elements of the cascade generalize across the FTLD spectrum. The earliest biological markers, plasma GFAP and NfL, together with WM burden and cognitive performance, were similar in the two groups, suggesting that astrogliosis and neuroaxonal degeneration are shared early events across syndromes. In contrast, frontal and insular atrophy and the behavioral and functional measures were more pronounced in the cortical syndromes, reflecting their frontoinsular and behavioural predominance relative to the comparatively preserved frontoinsular cortex of CBS and PSP. From a diagnostic standpoint, this indicates that the plasma biomarkers, precisely because they are similarly altered across syndromes, are sensitive markers of the underlying neurodegenerative process and are therefore well suited to early detection and staging, but are not on their own sufficient for the differential diagnosis of a specific FTLD‐associated syndrome, which continues to rely on clinical phenotyping and regional imaging. Nonetheless, because the cohort is predominantly composed of bvFTD and PPA cases, and the smaller numbers of CBS, PSP, and FTD‐ALS cases precluded standalone cascade analyses, the early positions of GFAP and TMT‐B are most firmly established for the cortical phenotypes; and broader generalization across the entire FTLD spectrum requires confirmation in adequately powered, syndrome‐specific cohorts.

Our findings both align with and diverge from the multimodal disease‐progression model of familial FTD reported by Staffaroni et al.,^43^ which modeled *C9orf72*, *GRN*, and *MAPT* variant carriers and non‐carrier controls. In that genetically defined cohort, regional atrophy and plasma NfL were among the earliest changes, emerging up to several decades before expected onset and in genotype‐specific orderings, with neuropsychological changes appearing closer to onset. Our cohort instead identified plasma GFAP and executive dysfunction (TMT‐B) as the earliest abnormalities. These differences likely reflect that autosomal‐dominant FTD shows genotype‐specific, decades‐long presymptomatic atrophy and early NfL elevation that need not generalize to sporadic disease; that Staffaroni et al. reported genotype‐specific orderings rather than a single cascade, whereas we pooled clinical syndromes; and that the biomarker panels differ, as we included plasma GFAP and TMT‐B (neither modeled previously), with GFAP departing early owing to its gradual trajectory and executive dysfunction being an early feature of the predominant cortical phenotypes. Within our cohort, excluding the 104 pathogenic variant carriers did not materially alter the biomarker ordering, indicating that the cascade is not driven by genetically determined cases.

These findings have implications for clinical practice and trial design. The early departure of GFAP and the early inflection of TMT‐B suggest that combining plasma astrogliosis markers with executive function testing could provide sensitive screening tools for early FTD. The differential transition dynamics imply that different biomarkers have optimal sensitivity at different stages, with plasma markers for prevention trials targeting early disease. The phenotype‐specific cascade orderings support the need for phenotype‐stratified trial designs, as the same biomarker may occupy different positions in the cascade depending on the clinical variant.

Several limitations should be acknowledged. First, the estimation of preclinical biomarker trajectories is inherently constrained by the study design: because most patients enter the cohort at or after symptom onset, data density in the pre‐symptomatic epochs is sparse, and the sigmoid trajectories in this window are driven more by parametric assumptions than by direct empirical observations. The confidence intervals for early‐transitioning biomarkers in the preclinical epochs are correspondingly wide, and prospective studies with known onset times would strengthen temporal precision. Additional limitations include the single‐center design, and the possibility that individual‐level trajectories may differ from the group‐level model.

In conclusion, this study provides the first biomarker cascade model for sporadic FTLD‐associated syndromes, revealing that astrogliosis and executive dysfunction are the earliest model‐estimated changes, followed by WM alterations, cortical atrophy centered on the insula, and clinical decline. These findings provide a framework for understanding disease mechanisms and designing clinical trials in FTLD‐associated syndromes.

## AUTHOR CONTRIBUTIONS

A.B. and B.B. conceived and designed the study. A.B. and B.B. performed the statistical analysis and wrote the first draft. A.B., V.B., E.P., V.C., F.P., A.S., M.S.C., G.B., R.M., A.A., R.G., and B.B. contributed to data acquisition. E.P., V.B., and R.A. contributed to MRI analysis. N.J.A., H.Z., and K.B. contributed to plasma biomarker analysis. All authors contributed to data interpretation, critically revised the manuscript for important intellectual content, and approved the final version. A.B. and B.B. had full access to all data in the study and had final responsibility for the decision to submit for publication. A.B. and B.B. have directly accessed and verified the underlying data reported in the manuscript.

## CONFLICT OF INTEREST STATEMENT

Alberto Benussi has received speaker honoraria from Angelini Pharma, Eisai, Eli Lilly and Novo Nordisk; he received research grants from Airalzh, Fondazione Cariplo, the Fondation pour la Recherche sur Alzheimer, and the Italian Ministry of University & Research; and he is listed as an inventor on issued patents on the use of non‐invasive brain stimulation for the differential diagnosis of dementia and to increase cognitive functions in patients with neurodegenerative disorders. Henrik Zetterberg has served at scientific advisory boards and/or as a consultant for Abbvie, Acumen, Alector, Alzinova, ALZpath, Amylyx, Annexon, Apellis, Artery Therapeutics, AZTherapies, Cognito Therapeutics, CogRx, Denali, Eisai, Enigma, LabCorp, Merck Sharp & Dohme, MerryLife, Nervgen, Novo Nordisk, Optoceutics, Passage Bio, Pinteon Therapeutics, Prothena, Quanterix, RedAbbeyLabs, reMYND, Roche, Samumed, ScandiBio Therapeutics AB, Siemens Healthineers, Triplet Therapeutics, and Wave. He has given lectures sponsored by Alzecure, BioArctic, Biogen, Cellectricon, Fujirebio, LabCorp, Lilly, Novo Nordisk, Oy Medix Biochemica AB, Roche, and WebMD. He is a co‐founder of Brain Biomarker Solutions in Gothenburg AB, which is part of the GU Ventures Incubator Program, and a shareholder of CERimmune Therapeutics (all outside the submitted work). Kaj Blennow. has served as a consultant and on advisory boards for Abbvie, AC Immune, ALZPath, AriBio, BioArctic, Biogen, Eisai, Lilly, Moleac Pte. Ltd, Novartis, Ono Pharma, Prothena, Roche Diagnostics, and Siemens Healthineers; has served on data‐monitoring committees for Julius Clinical and Novartis; has delivered lectures, produced educational materials, and participated in educational programs for AC Immune, Biogen, Celdara Medical, Eisai and Roche Diagnostics; and is a co‐founder of Brain Biomarker Solutions in Gothenburg AB, which is a part of the GU Ventures Incubator Program, outside the work presented in this article. Barbara Borroni has served at scientific advisory boards for Alector, Alexion/Astrazeneca, AviadoBio, Lilly, Denali, Wave, UCB. Valeria Bracca, Enrico Premi, Valentina Cantoni, Federica Palacino, Aurora Saccavini, Maria Sofia Cotelli, Giuliano Binetti, Rosa Manenti, Antonella Alberici, Roberto Gasparotti, Nicholas J Ashton, and Roberta Ghidoni declare no competing interests. Author disclosures are available in the Supporting Information.

## CONSENT STATEMENT

All participants, or their caregivers or legal representatives, provided written informed consent in accordance with the Declaration of Helsinki. The study protocol was approved by the Brescia Ethics Committee.

## Supporting information



Supporting Information

## Data Availability

De‐identified participant data, including clinical, imaging, and plasma biomarker data, will be made available to qualified researchers upon reasonable request to the corresponding author (B.B.).
