# Supplementary material for: Temporal order of clinical, imaging, and biomarker changes in frontotemporal lobar degeneration–associated syndromes
Source: Alzheimers Dement. 2026 Aug 5;22(8):e71722. doi: 10.1002/alz.71722 (PMC13439745; doi:10.1002/alz.71722)
Supplement: Supplementary file 1 [file ALZ-22-e71722-s001.docx]

**Statistical analysis.**

Biomarker cascade modelling was designed to estimate the relative temporal ordering of multimodal biomarkers across the frontotemporal dementia (FTD) disease continuum in a pooled FTD spectrum cohort while accounting for repeated within-patient measurements, covariates, uncertainty in symptom-onset timing, heterogeneity in trajectory shape, and uncertainty in biomarker scaling. The primary estimand for cascade ordering was the biomarker-specific inflection time θ_k_, defined as the disease time point at which the modelled biomarker trajectory reached 50% of its estimated transition on a bounded abnormality scale. Smaller values of θ_k_ were interpreted as earlier transition within the pooled spectrum cohort. Because healthy-control data (n=27) were available for plasma neurofilament light chain (NfL) and glial fibrillary acidic protein (GFAP), and externally derived normative z-scores adjusted for age, sex, and education were available for Trail Making Test part A (TMT-A) and part B (TMT-B), calibrated secondary analyses were prespecified to improve the biological and clinical interpretability of abnormality for these measures while preserving a fully internally standardized primary analysis for cross-domain comparability.

The study sample comprised 489 patients with a clinical diagnosis within the FTD spectrum. Patients underwent up to five longitudinal assessments (T0 through T4) including plasma biomarker collection, structural MRI acquisition, and standardized neuropsychological testing. Of the 489 patients, 133 contributed one visit, 126 contributed two visits, 96 contributed three visits, 110 contributed four visits, and 24 contributed five visits, for a total of 1,904 patient-visit observations. Disease duration was defined as elapsed time in years from reported symptom onset to each assessment. Because symptom onset in FTD is vulnerable to recall error, informant dependence, and phenotype-related variability in symptom recognition, uncertainty in onset timing was incorporated through prespecified perturbation analyses embedded within the bootstrap pipeline.

Ten biomarkers were included across plasma, structural MRI, and neuropsychological or clinical domains: GFAP and NfL in plasma, white matter lesion volume, insula volume, frontal volume, and grey matter volume from structural MRI, and TMT-A, TMT-B, the Frontal Behavioural Inventory (FBI), and the Clinical Dementia Rating Sum of Boxes (CDR-SoB). Natural logarithmic transformation was applied before standardization for GFAP, NfL, white matter lesion volume, TMT-A, and TMT-B. For insula, frontal and grey matter volumes, signs were inverted after standardization so that larger standardized values consistently reflected greater pathological deviation.

Three normalization frameworks were prespecified. The primary framework used an internal symptomatic reference for all biomarkers in order to maximize cross-domain scaling consistency. Patients were ranked by baseline CDR-SoB and those in the first quartile (Q1) were designated as the internal reference group representing the mild end of the symptomatic spectrum. In this primary framework, FBI and, for comparability, also TMT-A and TMT-B were standardized using regression-based correction within the Q1 subgroup, whereas MRI biomarkers and CDR-SoB were standardized by the Q1 mean and standard deviation. A secondary plasma-calibrated framework used healthy-control-based normative normalization for NfL and GFAP while retaining internal Q1 standardization for all remaining biomarkers. A secondary multimodal-calibrated framework used healthy-control-based normalization for NfL and GFAP and externally derived age-, sex-, and education-adjusted normative z-scores for TMT-A and TMT-B while retaining internal Q1 standardization for MRI biomarkers, FBI, and CDR-SoB. The calibrated frameworks were interpreted as biological and clinical calibration analyses for biomarker placement rather than direct replacements for the primary internally standardized multimodal cascade because the biomarkers were not all referenced to a single common external source. In addition, a robust internal standardization using the median and median absolute deviation (MAD, scaled by 1.4826) was prespecified as a sensitivity framework.

For internally standardized neuropsychological measures, each biomarker was regressed within the Q1 reference group on age at examination, years of formal education, and sex, and standardized residual scores in the full cohort were computed as z_ijk_ = (y_ijk_ - ŷ^(Q1)^_ijk_) / σ̂_resid,Q1,k_, where ŷ^(Q1)^_ijk_ was the predicted value from the Q1 regression given the patient demographics and σ̂_resid,Q1,k_ was the residual standard deviation in Q1. For MRI biomarkers and CDR-SoB, internal z-scores were computed as z_ijk_ = (x_ijk_ - μ_Q1,k_) / σ_Q1,k_, where x_ijk_ was the observed or log-transformed value and μ_Q1,k_ and σ_Q1,k_ were the Q1 mean and standard deviation with Bessel correction.

For NfL and GFAP in the calibrated frameworks, healthy-control-based normative z-scores were derived after natural logarithmic transformation. Letting *h* index healthy-control participants and k ∈ {NfL, GFAP}, normative models were fitted as log(Y_hk_) = η_0k_ + g_k_(age_h_) + η_1k_sex_h_ + ε_hk_, where g_k_(·) denoted the age effect, modelled linearly in the primary healthy-control normative analysis and with spline alternatives in sensitivity analyses, and sex was coded as 0 for female and 1 for male. For each patient visit, healthy-control-based z-scores were computed as z^(HC)^_ijk_ = [log(Y_ijk_) - loĝ(Y)^(HC)^_ijk_] / σ̂_resid,HC,k_, where loĝ(Y)^(HC)^_ijk_ was the predicted healthy-control normative value given patient age and sex, and σ̂_resid,HC,k_ was the residual standard deviation from the healthy-control model. Additional healthy-control normative sensitivity analyses evaluated spline versus linear age effects, robust regression, residual MAD-based scaling, and age-by-sex interaction terms when healthy-control sample size supported stable estimation. In the plasma-calibrated normalization framework, the healthy-control-based normative models for GFAP (HC model R^2^ = 0.28, *n*_HC_ = 27) and NfL (HC model R^2^ = 0.62, *n*_HC_ = 27) produced inflection time estimates of θ_GFAP,HC_ = 1.55 years (Δθ = -0.50 from primary) and θ_NfL,HC_ = 3.50 years (Δθ = +0.22 from primary). These shifts did not alter the cascade ordering, with both biomarkers remaining in similar relative positions, confirming that the primary internal Q1 normalization and the HC-calibrated framework yielded concordant results for plasma biomarker placement.

For TMT-A and TMT-B in the multimodal-calibrated framework, externally derived normative z-scores adjusted for age, sex, and education were used directly and were not re-standardized using the internal Q1 FTD subgroup. Directionality was harmonized so that larger values represented greater pathological deviation. Concordance of TMT-A and TMT-B placement between internal and external scaling was assessed by comparing inflection estimates, bootstrap rank distributions, and pairwise ordering probabilities.

For each biomarker and each normalization framework, a covariate-adjusted longitudinal trajectory was estimated as a function of disease duration using mixed-effects models accounting for repeated within-patient observations. The primary stage 1 model was z_ijk_ = α_0k_ + f_k_(t_ij_) + γ_1k_age_ij_ + γ_2k_sex_i_ + δ_k_phenotype_i_ + h_k_(t_ij_, phenotype_i_) + b_0ik_ + b_1ik_t_ij_ + ε_ijk_, where z_ijk_ denoted the standardized biomarker value for patient *i* at visit *j*, t_ij_ denoted disease duration in years from symptom onset, f_k_(t) was a restricted cubic spline for disease duration with 3 degrees of freedom in the primary analysis, age_ij_ was age at examination, sex_i_ was sex, phenotype_i_ denoted clinical phenotype, δ_k_ represented phenotype main effects, h_k_(t_ij_, phenotype_i_) represented a prespecified low-complexity phenotype-by-time interaction term used to absorb major pooled heterogeneity without stratification, b_0ik_ and b_1ik_ were patient-specific random intercept and random slope terms, and ε_ijk_ was the residual error. To avoid overparameterization, the phenotype-by-time component was implemented as either a phenotype-specific linear deviation in time slope or a reduced-rank interaction structure (prespecified before analysis) rather than full phenotype-specific spline trajectories, and if model instability or convergence failure occurred, the interaction term was removed while retaining phenotype main effects. This approach preserves the pooled analysis while reducing bias from systematic phenotype differences in baseline level and progression shape. Random slope models were attempted by default and retained when convergence was achieved and variance estimates were nondegenerate; otherwise random-intercept-only models were used. Estimation used restricted maximum likelihood. Wald-type inference on fixed effects was used descriptively, and the principal purpose of this stage was trajectory estimation for downstream cascade fitting rather than hypothesis testing. Because age at examination and disease duration are related, sensitivity analyses replaced age at examination with age at onset, defined as age at examination minus disease duration.

Potential bias from informative missingness and noncompletion was addressed in both diagnostic and sensitivity analyses. The primary analysis assumed missing at random conditional on observed covariates and observed longitudinal history. To examine violations of this assumption, visit-level and test-level inverse probability weighting (IPW) sensitivity analyses were performed using logistic models for observation or completion that included disease duration, age, sex, phenotype, prior observed biomarker values when available, and prior visit completion status when available. Extreme weights were truncated at prespecified percentiles, at the 1st and 99th percentiles, and weighted stage 1 trajectories were propagated through subsequent stages. In addition, pattern-based diagnostic analyses compared estimated trajectories across completion pattern classes, such as patients with sparse follow-up versus those with sustained follow-up, to assess whether apparent late-stage behaviour could be materially explained by dropout patterns. Mean inverse probability weights ranged from 1.93 (FBI) to 7.50 (GFAP), with weights truncated at 20 to limit the influence of extreme inverse probabilities. After IPW reweighting, the cascade ordering was preserved. The largest θ shifts under IPW occurred for neuropsychological measures (TMT-B: -1.68 years; FBI: -1.63 years; TMT-A: -1.39 years), suggesting that informative dropout preferentially affects cognitively impaired patients at later disease stages, pulling the estimated inflection times slightly earlier when missingness is accounted for. By contrast, MRI biomarkers showed minimal IPW sensitivity (Δθ < 0.16 years for all four measures).

The estimated stage 1 marginal trajectory for each biomarker, denoted m̂_k_(t), was transformed to a bounded abnormality function B̃_k_(t) ∈ [0, 1] over the prespecified disease time window. Scaling was based on the predicted marginal trajectory rather than pooled raw observations to reduce sensitivity to unequal visit density, repeated-measure weighting, and extreme observed values. Specifically, B̃_k_(t) = [m̂_k_(t) - L_k_] / [U_k_ - L_k_], where L_k_ and U_k_ were the 5th and 95th percentiles of predicted marginal trajectory values evaluated over a dense time grid in the analysis window. Values were truncated to the interval from 0 to 1, and sensitivity analyses used 10th and 90th percentile anchors. Because downstream sigmoid approximation assumes monotone progression, monotonicity of m̂_k_(t) over the analysis window was evaluated numerically using grid-based first differences and, when available, bootstrap derivative summaries. A biomarker was flagged as nonmonotonic if a prespecified proportion of grid intervals (greater than 10 percent) showed direction reversal inconsistent with the expected pathological direction and the reversal persisted in a nontrivial fraction of bootstrap replicates. Flagged biomarkers were retained in the multimodal analysis but their sigmoid parameters were interpreted explicitly as descriptive compression of the trajectory rather than evidence of a biologically sigmoidal transition, and robustness claims about their precedence were downgraded. Eight of ten biomarkers showed monotonically increasing bounded trajectories over the analysis window. GFAP and NfL were flagged as nonmonotonic based on their LME marginal trajectories. In both cases, the nonmonotonicity was minor (maximum decrease per time step < 0.001 in normalized units), and the sigmoid summaries remained interpretable as descriptive compressions of the overall trajectories rather than evidence of a sharply sigmoidal transition. Precedence claims involving GFAP and NfL were accordingly interpreted with the caveat that their trajectories did not exhibit strong monotone progression in the linear mixed-effects marginal fit.

Each bounded trajectory B_k_(t) was summarized by a biomarker-specific logistic sigmoid B_k_(t) = 1/[1 + exp(-β_k_(t - θ_k_))], where θ_k_ was the inflection time used for cascade ordering and β_k_ > 0 was the biomarker-specific steepness. Parameters were estimated jointly using weighted penalized least squares on a dense disease time grid t_g_, minimizing Σ_g_ w_kg_[B̃_k_(t_g_) - B_k_(t_g_; θ_k_, β_k_)]^2^ + λ(β_k_ - β_0_)^2^, where β_0_ = 0.15 was the regularization centre and λ was a prespecified penalty strength held constant across biomarkers in the primary analysis. To avoid ambiguity in weighting, inverse-variance weights were derived for all biomarkers from the estimated uncertainty of the stage 1 marginal trajectory on the grid using the model-based prediction variance (including fixed-effect uncertainty and, where implemented, bootstrap-based smoothing of prediction variance), and weights were rescaled within biomarker to have mean 1 for numerical stability; equal weights were used only in a prespecified sensitivity analysis. Parameter constraints were θ_k_ ∈ [-8, 18] years and β_k_ ∈ [0.03, 1.0]. The primary cascade ordering was determined by ranked θ_k_ values. Weighted residual sum of squares and descriptive R^2^ on the bounded predicted trajectory were computed to evaluate how well the sigmoid summarized the estimated trajectory. Because θ_k_ and β_k_ may be weakly identifiable for shallow or approximately linear trajectories, identifiability diagnostics were prespecified, including bootstrap dispersion of β_k_, sensitivity of θ_k_ rank to changes in λ, and local curvature of the objective function near the optimum.

Uncertainty in θ_k_, β_k_, and biomarker ranking was quantified using a patient-level cluster bootstrap with 1,000 iterations. In each replicate, patients were resampled with replacement and all visits for each selected patient were retained. The entire analysis pipeline was repeated in each replicate, including normalization according to the selected framework, stage 1 mixed-effects trajectory estimation, stage 2 bounded transformation, and stage 3 sigmoid fitting. Bootstrap standard errors were computed as the standard deviation of replicate estimates and 95 percent confidence intervals were defined by empirical 2.5th and 97.5th percentiles. To quantify uncertainty in relative ordering, for every biomarker pair (k, ℓ) the pairwise precedence probability P(θ_k_ < θ_ℓ_) was computed as the proportion of bootstrap replicates in which biomarker *k* preceded biomarker *ℓ*. Pairwise ordering probabilities were summarized in a heatmap. For each biomarker, the empirical bootstrap distribution of rank position from earliest (1) to latest (10) was summarized by median rank and interquartile range. Pointwise confidence bands for trajectories and derivatives were interpreted as uncertainty in the fitted summary under the specified modelling pipeline rather than as direct confidence bands for an unobserved true biological trajectory. Of the 45 unique biomarker pairs, 41 (91%) had pairwise precedence probabilities meeting the P ≥ 0.80 robustness criterion, indicating that the overall cascade ordering was well determined by the data. The four pairs that did not reach robustness involved biomarkers with closely spaced inflection times: WM Lesion Volume and GFAP (P = 0.54), Insula Atrophy and NfL (P = 0.69), NfL and Frontal Atrophy (P = 0.71), and Frontal Atrophy and GM Atrophy (P = 0.65). For all other pairs, the precedence probability was ≥ 0.80, and for most distal comparisons (such as TMT-B before CDR-SoB, or GFAP before FBI) the probability was 1.00. The complete pairwise precedence matrix is shown in **Supplementary Figure 1**.

Sensitivity analyses were prespecified to assess robustness to temporal anchoring, normalization, model specification, and sigmoid regularization. Symptom-onset uncertainty was evaluated by perturbing onset dates within each bootstrap replicate using mean-zero random error terms with prespecified standard deviations corresponding approximately to 0.5, 1, and 2 years, recomputing disease durations, and rerunning the full pipeline. Stability was assessed by changes in θ_k_, pairwise precedence probabilities, and rank distributions. Longitudinal trajectory sensitivity analyses varied spline flexibility (2, 3, and 4 degrees of freedom), covariate parameterization (age at examination versus age at onset), and random effects structure (random intercept only versus random intercept plus slope, subject to convergence). Sigmoid sensitivity analyses varied the penalty strength λ, the regularization centre β_0_ (for example 0.10, 0.15, and 0.20), and the stage 2 anchor percentiles (predicted trajectory P_10_ and P_90_ instead of P_5_ and P_95_), and included a comparison model with fixed steepness β_k_ = 0.15 for all biomarkers. Normalization sensitivity analyses compared the primary internal Q1 framework, the plasma-calibrated framework, the multimodal-calibrated framework, and the robust internal median-and-MAD framework. For NfL and GFAP, concordance between internal and healthy-control-based normalization was assessed by comparing inflection estimates, median rank, and pairwise ordering probabilities versus all other biomarkers. For TMT-A and TMT-B, concordance between internal and external normative scaling was assessed similarly. The cascade ordering was highly stable across all prespecified sensitivity conditions (**Supplementary Figure 2**). Onset-date perturbation with standard deviations of 0.5, 1.0, and 2.0 years produced identical biomarker orderings, with mean θ_k_ shifts never exceeding 0.35 years even at σ = 2.0 years (range of SD across biomarkers: 0.11-0.15 years). Sigmoid regularization sensitivity analyses (varying β_0_ across 0.10, 0.15, 0.20, and 0.25) produced maximum θ shifts of up to 2.9 years for individual biomarkers, but the overall ordering remained stable. The IPW analysis produced identical ordering. The largest IPW-induced θ shifts occurred for TMT-B (-1.68 years) and FBI (-1.63 years), consistent with greater informative dropout in neuropsychological testing.

To reduce overinterpretation of a single ranked list, robustness of ordering claims was prespecified. A pairwise precedence claim was considered robust only if the pairwise precedence probability exceeded a high threshold (at least 0.80 or at most 0.20 for reversed precedence) and the direction of precedence was stable across key sensitivity analyses, including onset perturbation, normalization framework, and spline flexibility. The primary reported results were based on the internally standardized framework and included biomarker-specific θ_k_ and β_k_ estimates with 95 percent confidence intervals, rank distributions, pairwise precedence probability matrices, and bounded trajectory and sigmoid plots with bootstrap confidence bands. Secondary results from the plasma-calibrated and multimodal-calibrated frameworks reported changes in placement of NfL and GFAP and, in the multimodal-calibrated framework, TMT-A and TMT-B, together with changes in pairwise precedence probabilities and rank distributions relative to the primary analysis, and were interpreted as biological and clinical calibration analyses rather than replacements of the primary cascade.

For inferential trajectory summaries, the instantaneous rate of change was derived from the estimated biomarker-specific sigmoid parameters as Sk(t) = dBk/dt = βkBk(t)[1 - Bk(t)], with peak rate Sk,max = βk/4 occurring at t = θk. Pointwise 95 percent confidence bands for sigmoid trajectories and derivative curves were obtained from bootstrap replicates by evaluating Bk(t; θ(b)k, β(b)k) on a dense time grid and taking empirical percentiles at each time point.

To assess whether the cascade ordering was influenced by the inclusion of genetically determined cases, the entire pipeline (internal first-quartile standardization, logistic-sigmoid inflection-time estimation, and patient-level cluster bootstrap with 1,000 iterations) was repeated after excluding all carriers of a pathogenic variant (n = 104), retaining the 385 non-carriers. Biomarker-specific inflection times (θ) and 95% confidence intervals were re-estimated, and concordance with the primary full-cohort ordering was quantified with the Spearman rank correlation of θ across biomarkers.

MRI field strength (1.5T or 3T) was recorded for every imaging time point (245 scans at 1.5T and 271 at 3T). Because images were acquired on both systems, all T1-weighted and FLAIR scans were processed with a single uniform pipeline (CAT12 for grey matter volumes and the Lesion Segmentation Toolbox for white matter hyperintensity volume), with identical segmentation, spatial normalization, modulation, and smoothing irrespective of field strength. Because 3T acquisitions were somewhat more frequent at later disease stages, an additional sensitivity analysis re-estimated the cascade after adjusting the MRI biomarkers (grey matter, frontal, and insular volumes and white matter lesion volume) for field strength while retaining disease duration; the biomarker ordering was essentially unchanged (Spearman ρ = 0.98), with differences limited to small shifts (< 0.7 years) among the closely-spaced cortical atrophy measures.

Analyses were conducted in Python 3.10 using NumPy, pandas, statsmodels for mixed-effects and regression models, SciPy for bounded optimization, and matplotlib for visualization.

**Supplementary Table 1. Genetic composition of the cohort: pathogenic variant carriers by clinical syndrome and gene.**

| **Syndrome** | **C9orf72** | **GRN** | **MAPT** | **TARDBP** | **FUS** | **SQSTM1** | **Carriers, n** |
| --- | --- | --- | --- | --- | --- | --- | --- |
| bvFTD | 20 | 28 | 3 | 5 | 0 | 0 | 56 |
| nfvPPA | 4 | 28 | 0 | 0 | 0 | 1 | 33 |
| svPPA | 0 | 1 | 0 | 1 | 0 | 0 | 2 |
| CBS | 0 | 4 | 1 | 0 | 0 | 0 | 5 |
| PSP | 0 | 0 | 0 | 0 | 0 | 0 | 0 |
| FTD-ALS | 5 | 0 | 0 | 2 | 1 | 0 | 8 |
| Total | 29 | 61 | 4 | 8 | 1 | 1 | 104 |

Values are counts of pathogenic variant carriers. Percentages are the proportion of carriers within each syndrome. bvFTD, behavioural variant frontotemporal dementia; nfvPPA/svPPA, non-fluent/semantic variant primary progressive aphasia; CBS, corticobasal syndrome; PSP, progressive supranuclear palsy; FTD-ALS, frontotemporal dementia with amyotrophic lateral sclerosis.

**Supplementary Table 2. Number of available observations for each biomarker, overall and by epoch (years from symptom onset).**

| **Biomarker** | **Obs (N)** | **Patients (n)** | **<0** | **0–5** | **5–10** | **10–15** | **>15** |
| --- | --- | --- | --- | --- | --- | --- | --- |
| Plasma GFAP | 370 | 217 | 47 | 194 | 68 | 48 | 13 |
| Plasma NfL | 372 | 219 | 47 | 195 | 69 | 48 | 13 |
| WMH lesion volume | 465 | 310 | 42 | 288 | 77 | 48 | 10 |
| Insula volume | 634 | 396 | 45 | 409 | 112 | 58 | 10 |
| Frontal volume | 634 | 396 | 45 | 409 | 112 | 58 | 10 |
| Grey matter volume | 633 | 396 | 45 | 408 | 112 | 58 | 10 |
| TMT-A | 827 | 368 | 50 | 540 | 165 | 61 | 11 |
| TMT-B | 709 | 328 | 50 | 456 | 136 | 56 | 11 |
| FBI | 1044 | 441 | 60 | 693 | 216 | 64 | 11 |
| CDR-SoB | 823 | 448 | 54 | 545 | 156 | 58 | 10 |

Epochs are defined by disease duration (years from symptom onset). Counts reflect non-missing observations. WMH, white matter hyperintensity; GFAP, glial fibrillary acidic protein; NfL, neurofilament light chain; TMT, Trail Making Test; FBI, Frontal Behavioural Inventory; CDR-SoB, Clinical Dementia Rating Sum of Boxes.

**Supplementary Table 3. Biomarker inflection times (θ) in the full cohort and after excluding pathogenic variant carriers.**

| **Biomarker** | **Full cohort θ [95% CI]** | **Rank** | **Non-carriers θ [95% CI]** | **Rank** |
| --- | --- | --- | --- | --- |
| TMT-B | 0.28 [-0.74, 1.32] | 1 | 0.61 [-0.49, 1.64] | 1 |
| WMH lesion volume | 1.96 [1.14, 2.82] | 2 | 1.22 [0.28, 2.17] | 2 |
| GFAP | 2.06 [0.94, 3.14] | 3 | 1.22 [-0.16, 2.66] | 3 |
| Insula atrophy | 2.88 [2.02, 3.80] | 4 | 3.73 [2.74, 4.85] | 6 |
| NfL | 3.28 [1.98, 4.55] | 5 | 3.69 [2.38, 4.97] | 4 |
| Frontal atrophy | 3.75 [2.82, 4.61] | 6 | 3.76 [2.74, 4.79] | 7 |
| GM atrophy | 3.95 [3.09, 4.90] | 7 | 3.73 [2.79, 4.77] | 5 |
| TMT-A | 5.79 [4.99, 6.65] | 8 | 6.06 [5.15, 6.98] | 8 |
| FBI | 7.05 [6.23, 7.94] | 9 | 7.76 [6.83, 8.74] | 9 |
| CDR-SoB | 7.89 [6.98, 8.72] | 10 | 8.00 [7.06, 8.95] | 10 |

θ, inflection time (years from symptom onset); 95% confidence intervals from a 1,000-iteration patient-level cluster bootstrap. Full cohort, n = 489; non-carriers, n = 385. Spearman rank correlation of θ between the two analyses = 0.93. Biomarkers are ordered by full-cohort θ.

**Supplementary Figure 1. Pairwise precedence probability matrix for the estimated biomarker cascade ordering.**

Legend: Each cell shows the bootstrap-derived probability P(θ_row < θ_col) that the row biomarker reaches 50% abnormality before the column biomarker, computed across 1,000 patient-level cluster bootstrap replicates. Values are color-coded on a diverging scale from blue (P = 0, column precedes row) through white (P = 0.50, indeterminate) to red (P = 1.00, row precedes column). Bold values indicate robust pairwise ordering claims (P ≥ 0.80 or P ≤ 0.20). Biomarkers are arranged from earliest (top/left) to latest (bottom/right) inflection time. Coloured side strips denote biomarker domain: red = blood biomarkers, blue = MRI, green = neuropsychological/clinical. Of 45 unique pairs, 41 (91%) met the robustness threshold; the four non-robust pairs involved biomarkers with closely spaced inflection times (WMH Lesion Volume-GFAP, Insula Atrophy-NfL, NfL-Frontal Atrophy, Frontal Atrophy-GM Atrophy).

**Supplementary Figure 2. Sensitivity of cascade ordering to analytic choices.**

Legend: Forest plot showing estimated inflection times (θ, in years from symptom onset) for each biomarker under different analytic conditions. Biomarkers are ordered from earliest (top) to latest (bottom) inflection time. Black circles represent the primary analysis estimate (β₀ = 0.15) with 95% bootstrap confidence intervals. Red squares indicate sigmoid regularization sensitivity analyses (β₀ = 0.10, 0.20, 0.25). Purple diamonds show inverse-probability-weighted (IPW) estimates adjusting for informative missingness. Blue upward triangles show mean θ under symptom-onset date perturbation (σ = 0.5, 1.0, 2.0 years). Green downward triangles show θ from healthy-control-calibrated normalization (NfL and GFAP only). Row background shading indicates biomarker domain: red = blood biomarkers, blue = MRI, green = neuropsychological/clinical. The cascade ordering was highly stable across all conditions, with onset perturbation producing identical orderings.

**Supplementary Figure 3. Sensitivity of the biomarker cascade to exclusion of pathogenic variant carriers.**

Legend: Biomarker-specific inflection times (θ, years from symptom onset) with 95% confidence intervals are shown for the full cohort (n = 489; circles) and after excluding all 104 pathogenic variant carriers (n = 385; squares), ordered by full-cohort θ. Confidence intervals overlap for every biomarker and the ordering is essentially unchanged (Spearman ρ = 0.93), indicating that the early positions of plasma GFAP and executive dysfunction (TMT-B) are not driven by genetically determined cases. GFAP, glial fibrillary acidic protein; NfL, neurofilament light chain; WMH, white matter hyperintensity; TMT, Trail Making Test; FBI, Frontal Behavioural Inventory; CDR-SoB, Clinical Dementia Rating Sum of Boxes.

**Supplementary Figure 4. Adjusted comparison of baseline biomarkers between cortical (bvFTD, PPA; n=340) and motor-predominant (CBS, PSP, FTD-ALS; n=149) FTLD-associated syndromes.**

Legend: Points show the adjusted standardized difference (Motor − Cortical) with 95% confidence intervals, estimated from baseline linear regression models adjusting for disease duration, age, and sex; markers are coloured by biomarker domain. Values to the left of zero indicate greater abnormality in the cortical syndromes. **p*<0.05, ***p*<0.01, ****p*<0.001. GFAP, glial fibrillary acidic protein; NfL, neurofilament light chain; WMH, white matter hyperintensity; TMT, Trail Making Test; FBI, Frontal Behavioural Inventory; CDR-SoB, Clinical Dementia Rating Sum of Boxes.
